# The absolute and relative sizes of the brains and bodies of fetuses with different forms of congenital heart disease and intrauterine growth restriction

**DOI:** 10.1186/1532-429X-18-S1-P151

**Published:** 2016-01-27

**Authors:** Theo Kingdom, Meng Yuan Zhu, Prashob Porayette, Mike Seed, Brahmdeep S Saini, Ioana A Stochitoiu, Lars Grosse-Wortmann, Shi-Joon Yoo, Edgar Jaeggi, Christopher Macgowan, John Kingdom, Jessie Mei Lim

**Affiliations:** 1 SickKids Hospital, Toronto, ON Canada; 2 Mount Sinai Hospital, Toronto, ON Canada

## Background

Congenital heart disease(CHD) is associated with in utero brain dysmaturation, abnormal cerebral vasculature and decreased brain size.(1,2) Intrauterine growth restriction is associated with increased relative brain size, smaller birth weight and neurological impairment later in life.(3) A large cohort of CHD and IUGR fetuses allows for more definitive study as to the extent these conditions effect the absolute and relative sizes of the brain and body.

## Methods

The study involves 29 fetuses with Intrauterine Growth Restriction (IUGR), 127 fetuses with congenital heart disease (CHD) and 62 normal control fetuses. Of those 127 fetuses with CHD, 20 had transposition of the great arteries (TGA), 25 had coarctation (CoA), 22 had tetralogy of fallot (TOF), 37 had single ventricle hearts (SV) and 23 had other forms of CHD. A T2 trufi 3d maternal cor chop sequence was performed to measure fetal brain and body volume. Both were converted to estimated weights using published conversion algorithms.(4) The weights and relative weights of both the heart and brains of the groups and CHD subgroups were examined using unpaired t-tests to determine significance.

## Results

There were no significant differences between the corrected gestational ages of any of the groups.

Normal fetuses have higher brain weights than IGUR (p < 0.0001) and CHD fetuses (p=0.02) while CHD and IUGR fetuses are similar (p=0.1). Also, normal fetuses are heavier than fetuses with SV (p=0.005), TOF (p=0.008) and other diagnoses (p=0.046). IUGR fetuses are heavier than fetuses with TGA (p=0.03) and CoA (p=0.02). Differences with other CHD subgroups were insignificant.

Normal and CHD fetuses have larger fetal weights than IUGR fetuses (p < 0.0001, p < 0.0001) while normal and CHD fetuses are similar (p=0.9). All CHD subgroups have the same significance as the whole group. IUGR fetuses have larger brain to body weight ratios than Normal and CHD fetuses(including subgroups) (all p < 0.0001). Normal ratios are higher than CHD ratios (p=0.02). Also, The brain to body weight ratios of normal fetuses is higher than CHD fetuses with TGA (p=0.003), SV (p < 0.0001) and other diagnoses (p < 0.04).

## Conclusions

Normal fetuses have largest brains then CHD, then IUGR brains. Normal fetuses are larger than IUGR fetuses, but are similar size to CHD fetuses. In terms of brain weight to body weight ratio, IUGR babies have the largest brains for their body size, then normals, then CHD fetuses. When the CHD cohort is broken into 5 different CHD diagnoses, conclusions regarding fetal weight are unaffected. Regarding fetal brain volume, normal fetuses have the largest brains then fetuses with less serious forms of CHD(TGA, CoA), then fetuses with more serious forms of CHD(SV, TOF, Other). All CHD subgroups have significantly lower brain wieght to body weight ratios than IUGR babies. Normal babies only have significantly larger ratios than CHD babies with TGA, SV and other diagnoses.

**Table 1:** The absolute and relative sizes of the brains and bodies of fetuses with different forms of congenital heart disease and intrauterine growth restriction.

	Estimated Brain Weight (g)	Estimated Body Weight (kg)	Brain to Body Weight Ratio
Normal	304.71	2.812	0.109
IUGR	269.07	1.951	0.143
All CHD	287.01	2.804	0.104
TGA	297.55	2.981	0.101
SV	276.73	2.820	0.098
CoA	307.00	2.771	0.113
Other	282.27	2.778	0.103
TOF	276.95	2.681	0.104

**Figure 1:**
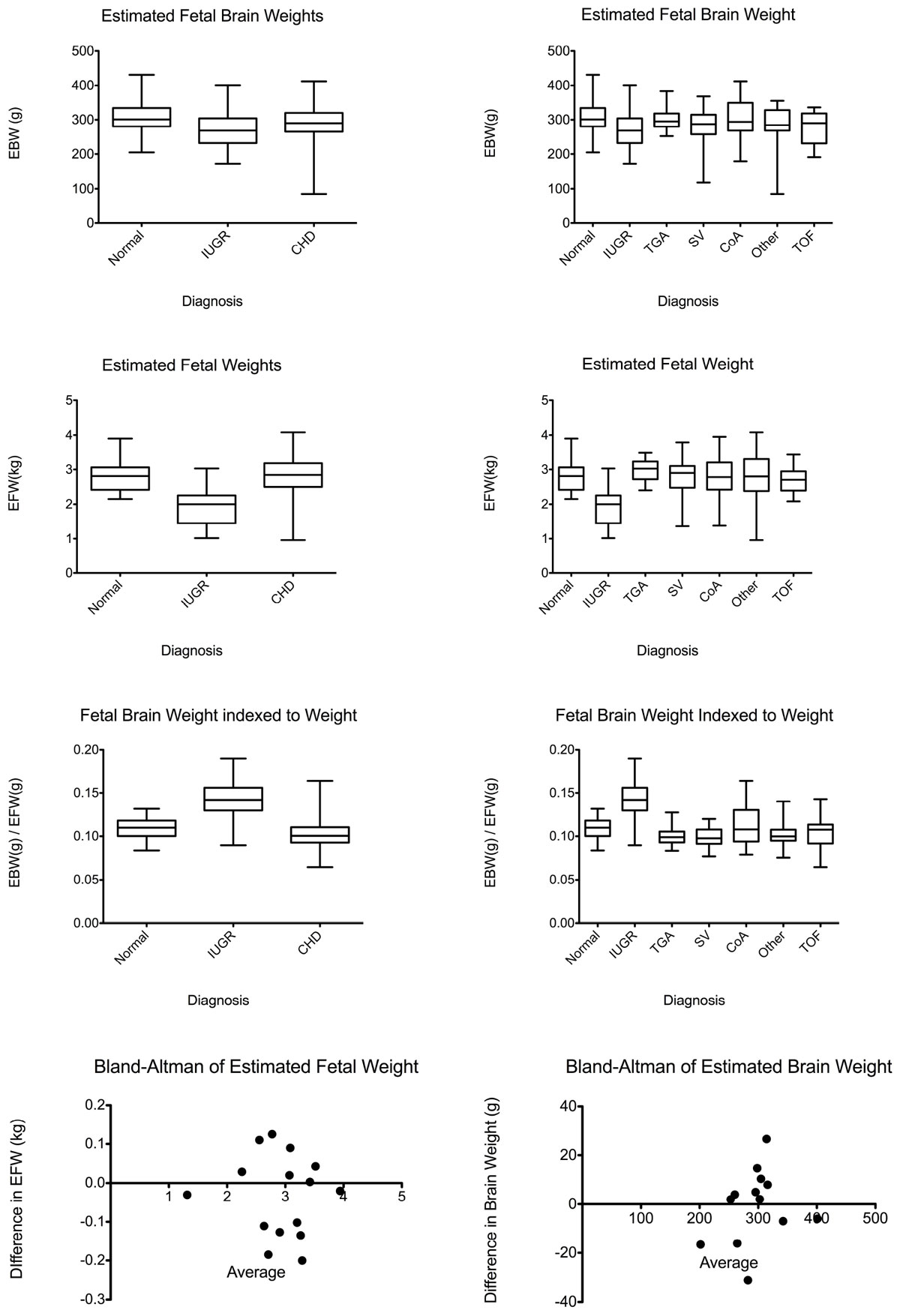
**The absolute and relative sizes of the brains and bodies of fetuses with different forms of congenital heart disease and intrauterine growth restriction**. (Normal n = 62, IUGR n = 29, CHD=125) (CHD: TGA n = 2-, CoA n = 25, TOF n = 20, SV n = 37, Other n = 23)
